# Comment on: "The role of EPAS-1 and ghrelin in right ventricular dysfunction in systemic lupus erythematosus"

**DOI:** 10.1590/1806-9282.20250796

**Published:** 2025-10-27

**Authors:** Selin Cilli Hayıroğlu

**Affiliations:** 1 Istanbul Goztepe Prof. Dr. Suleyman Yalcin City Hospital, Department of Rheumatology – Istanbul, Turkey.

Dear Editor,

I have read with great interest the recent article entitled "The Role of EPAS-1 and Ghrelin in Right Ventricular Dysfunction in Systemic Lupus Erythematosus."^1^ The study provides valuable insights into the complex cardiovascular manifestations of systemic lupus erythematosus (SLE) and highlights the potential role of novel biomarkers such as EPAS-1 and ghrelin. The thorough methodology, particularly the blinded echocardiographic assessments and careful exclusion criteria, lends credibility to the findings.

One of the strengths of this work is its focus on the right ventricular function, a relatively underexplored yet clinically significant aspect of SLE-associated cardiac involvement. The identification of elevated EPAS-1 and ghrelin levels and their potential links to right ventricular dysfunction opens promising avenues for further research into prognostic markers and therapeutic targets. Although we know that right ventricular function may vary when SLE presents with different non-cardiac manifestations, we have limited information regarding the markers that reveal right ventricular dysfunction^2^. Moreover, the presence of right ventricular dysfunction can be associated with a poor prognosis in patients with SLE, and predicting this early becomes important for the close monitoring of patients^3^.

However, certain limitations warrant consideration. The relatively small sample size (34 patients with SLE and 35 controls) may limit the generalizability of the results. Additionally, the cross-sectional design precludes the establishment of causal relationships between biomarker levels and cardiac function. Longitudinal studies would be instrumental in confirming whether EPAS-1 and ghrelin levels predict progression or improvement in right ventricular dysfunction over time. Moreover, while the study acknowledges the potential effects of medications such as hydroxychloroquine and corticosteroids on cardiac parameters, a more detailed subgroup analysis based on treatment regimens could have provided deeper insights into their modulatory roles, especially since corticosteroid use has been reported to have a positive effect on right ventricular function in patients with SLE^4^.

In conclusion, this study makes a meaningful contribution to the understanding of cardiovascular complications in SLE and proposes exciting new biomarkers for future investigation. Further larger, multi-center, and longitudinal studies are needed to validate and expand upon these important findings.

## Data Availability

The datasets generated and/or analyzed during the current study are available from the corresponding author upon reasonable request.
